# diff_classifier: Parallelization of multi-particle tracking video analyses

**DOI:** 10.21105/joss.00989

**Published:** 2019-04-10

**Authors:** Chad Curtis, Ariel Rokem, Elizabeth Nance

**Affiliations:** 1 Department of Chemical Engineering, University of Washington; 2 eScience Institute, University of Washington

## Abstract

The diff_classifier package seeks to address the issue of scale-up in multi-particle tracking (MPT) analyses via a parallelization approach. MPT is a powerful analytical tool that has been used in fields ranging from aeronautics to oceanography (Pulford, 2005) allowing researchers to collect spatial and velocity information of moving objects from video datasets. Examples include:

- Tracking tracers in ocean currents to study fluid flow
- Tracking molecular motors (e.g., myosin, kinesin) to assess motile activity
- Measuring intracellular trafficking by tracking membrane vesicles
- Assessing microrheological properties by tracking nanoparticle movement.

While a variety of tracking algorithms are available to researchers (Chenouard et al., 2014), a common problem is that data analysis usually depends on the use of graphical user interfaces, and relies on human input for accurate tracking. For example, particle detection often relies on the selection of a quality threshold, a numerical quantity distinguishing between “real” particles and “fake” particles (Tineves et al., 2017). If this threshold is too high, false positive trajectories result in skewed MSD profiles, and in extreme cases, cause the code to crash due to a lack of convergence in the particle linking step. If the threshold is too low, trajectories will be cut short resulting in a bias towards short fast-moving trajectories and could result in empty datasets (Wang, Nunn, Harit, McKinley, & Lai, 2015).

Due to variations in experimental conditions and image quality, user-selected tracking parameters can vary widely from video to video. As parameter selection can also vary from user to user, this also brings up the issue of reproducibility. diff_classifier addresses these issues with regression tools to predict input tracking parameters and parallelized script-based implementations in Amazon Web Services (AWS), using the Simple Storage Service (S3) and Batch for data storage and computing, respectively, and relying on the Cloudknot software library for automating these interactions (Richie-Halford & Rokem, 2018). By manually tracking a small subset of the entire video dataset to be analyzed (5–10 videos per experiment), users can predict tracking parameters based on intensity distributions of input images. This can simultaneously reduce time-to-first-result in MPT workflows and provide reproducible MPT results.

diff_classifier also includes downstream MPT analysis tools including mean squared displacement and feature calculations, visualization tools, and a principal component analysis implementation. MPT is commonly used to calculate and report ensemble-averaged diffusion coefficients of nanoparticles and other objects. We sought to expand the power of MPT analyses by changing the unit of analysis to individual particle trajectories. By including a variety of features (e.g., aspect ratio, boundedness, fractal dimension), with trajectory-level resolution, users can implement a range of data science analysis techniques to their MPT datasets.
